# Repair Versus Replacement in Mitral Valve Endocarditis Due to Methicillin-Susceptible *Staphylococcus aureus*

**DOI:** 10.3390/pathogens14090839

**Published:** 2025-08-23

**Authors:** Zaki Haidari, Iskandar Turaev, Stephan Knipp, Mohamed El-Gabry

**Affiliations:** Department of Thoracic and Cardiovascular Surgery, West German Heart and Vascular Centre, University Hospital Essen, Hufelandstrasse 55, 45147 Essen, Germany

**Keywords:** infective endocarditis, mitral valve, repair, *Staphylococcus aureus*

## Abstract

**Background:** The guidelines recommend mitral valve repair whenever possible in patients undergoing surgical treatment for active infective endocarditis of the native mitral valve. However, the impact of causative microorganisms in relation to treatment strategies, especially *Staphylococcus aureus*, has not been studied. In this study, we aimed to compare the outcomes of mitral valve repair versus replacement in patients with native mitral valve infective endocarditis caused by methicillin-susceptible *Staphylococcus aureus*. **Methods:** Consecutive patients with definitive active infective endocarditis of the native mitral valve caused by methicillin-susceptible *Staphylococcus aureus* undergoing cardiac surgery between 2012 and 2022 were selected. Patients were classified according to the treatment received in two groups: repair and replacement. Inverse propensity treatment weighting was employed to correct for confounders. The endpoints were all-cause mortality, incidence of recurrent endocarditis, reoperation rate, and event-free survival at two-year follow-up. **Results:** Among 170 operated-upon patients with active infective endocarditis of the native mitral valve, 44 cases were caused by methicillin-susceptible *Staphylococcus aureus*. A total of 23 patients underwent mitral valve repair and 21 patients received mitral valve replacement. Weighted 30-day mortality in the repair group was 43%, versus 27% in the replacement group (*p* = 0.15). Two-year mortality increased to 57% in the repair group and 32% in the replacement group (*p* = 0.02). Three patients developed recurrent endocarditis in the repair group, while no recurrent endocarditis occurred in the replacement group. Three patients in the repair group required reoperation due to recurrence and one patient in the replacement group underwent re-operation due to paravalvular leakage. Weighted two-year event-free survival was 29% in the repair group and 59% in the replacement group (*p* < 0.01). **Conclusions:** Mortality in patients with mitral valve infective endocarditis caused by *Staphylococcus aureus* is extremely high, especially in patients undergoing mitral valve repair. The risk of recurrent endocarditis and mid-term mortality seems to be higher in mitral valve repair, resulting in poor event-free survival during two-year follow-up. However, the sample size was likely insufficient for drawing definitive conclusions.

## 1. Introduction

Patients undergoing surgical treatment for active native mitral valve infective endo-carditis are at the highest risk of mortality among endocarditis patients [1]. The current guidelines recommend mitral valve repair whenever feasible [2]. Studies suggest that outcomes are better if the valve is repaired rather than replaced, with fewer reinfections and reoperations [3,4]. The most common causative microorganism in native mitral valve infective endocarditis is *Staphylococcus aureus* [5]. *Staphylococcus aureus* infective endocarditis seems to be associated with higher mortality and risk of recurrence compared to other pathogens [6,7,8,9]. However, the role of mitral valve repair in patients with native mitral valve infective endocarditis due to *Staphylococcus aureus* has not been studied. In this study, we compared the outcomes of valve repair and replacement in patients with native mitral valve infective endocarditis caused by *Staphylococcus aureus* undergoing surgical treatment.

## 2. Methods

### 2.1. Participants

Patients with active infective endocarditis of the native mitral valve undergoing cardiac surgery between 2012 and 2022 were eligible. The diagnosis was according to the modified Duke criteria [10]. The selected population consisted of patients with *Staphylococcus aureus*, confirmed via blood cultures. Patients with methicillin-resistant *Staphylococcus aureus* as the causative microorganism were excluded. Prospectively collected perioperative data were retrieved from the hospital’s electronic health record system. Follow-up data on mortality and valve-related complications were obtained via outpatient visits or by directly contacting the patients, referral cardiologists, and/or general physicians. The institutional ethics committee approved the study and written informed consent was signed by all participants.

### 2.2. Surgical Technique

Preoperatively, transesophageal echocardiography was used to assess cardiac function and the extension of infective endocarditis. The chest was accessed through median sternotomy. After heparinization, aortic and caval vessels were cannulated. Antegrade cold crystalloid cardioplegia (Custodiol, Dr. Franz Koehler Chemie, Bensheim, Germany) and topical cooling were used to achieve cardioplegic arrest and myocardial protection. The mitral valve was exposed either through left atriotomy or via the transseptal approach, depending on the necessity for the additional tricuspid procedure. A thorough and comprehensive valve analysis was performed, identifying the presence and location of infective and degenerative lesions. First, all vegetations were removed and all infected tissue was resected. The remaining valvular tissue was disinfected using polyvidone and vancomycin. The decision on whether to repair or replace the valve was at the discretion of the operating surgeon. In the repair group, the defects were either directly closed or patch plasty was performed. Standard repair techniques included chordal transfer or replacement. Finally, the repair was completed via annuloplasty. In the replacement group, mitral valve replacement was performed after the radical resection of infected tissue. Concomitant procedures were performed in patients with additional cardiac pathology. After weaning from cardiopulmonary bypass, transesophageal echocardiography was performed to evaluate the surgical result. Patients were transferred postoperatively to the cardiac surgical intensive care unit. The direct postoperative goals consisted of hemodynamic stabilization and early extubation. Intravenous antibiotic therapy continued for up to 6 weeks according to the guidelines. Before hospital transfer and discharge, transthoracic echocardiography was performed to evaluate cardiac and valvular function.

### 2.3. Outcome Measures

The primary endpoints were overall mortality, the incidence of recurrent endocarditis, and the reoperation rate during two-year follow-up. Recurrent endocarditis was defined as any repeat episode of infective endocarditis of the mitral valve, either by the same (relapse) microorganism or by a different (reinfection) microorganism during two-year follow-up. Reoperation was defined as any reoperation on the mitral valve. Secondary endpoints consisted of postoperative complications, intensive care unit, or hospital stay.

### 2.4. Statistical Analysis

SPSS software version 30 (SPSS Inc., Chicago, IL, USA) was used for statistical analysis. Continuous variables were expressed as the mean or median with standard deviation (SD) or interquartile range (IQR), as appropriate. Student’s *t*-test or the Mann–Whitney test was used to compare continuous variables. Categorical variables were presented as the absolute number of patients and frequencies. The chi-square test, including Yates’ correction for continuity, was used to compare categorical variables. The standardized mean difference (SMD) for each pre- and intraoperative variable was calculated to evaluate the balance between the two groups. An SMD value of less than 0.25 was considered an acceptable difference. Inverse propensity treatment weighting (IPTW) was performed to correct for imbalances between the two comparing groups. The IPTW model included dialysis, previous cardiac surgery, the European System for Cardiac Operative Risk Evaluation II, concomitant aortic valve endocarditis, concomitant tricuspid valve surgery, surgical indication, preoperative intubation, preoperative vasopressor need, isolated mitral valve surgery, concomitant tricuspid valve surgery, cardiopulmonary bypass, and aortic cross-clamp times. Unweighted and weighted analysis was performed to compare the two groups. A two-sided *p*-value of less than 0.05 was considered an indication of statistical significance.

## 3. Results

### 3.1. Baseline Characteristics

**Figure 1:**
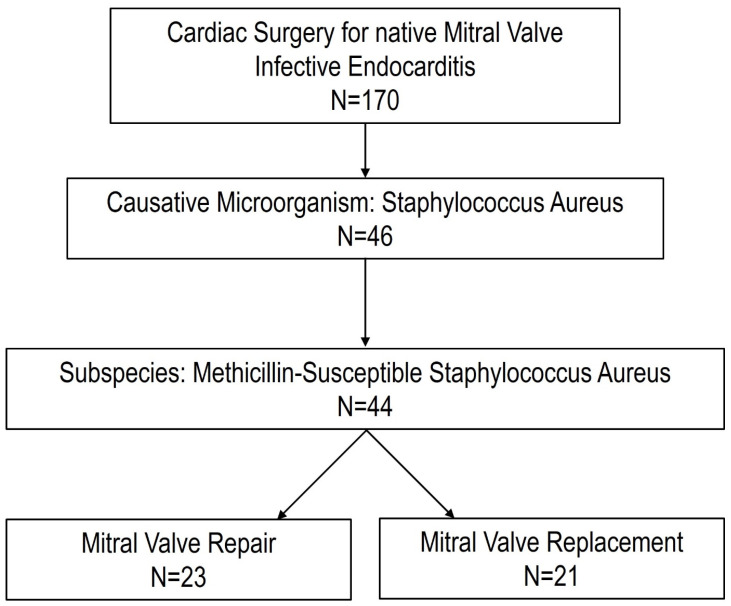
Flowchart of the study.

Between January 2012 and January 2022, 170 patients with native mitral valve infective endocarditis underwent surgical treatment. In 46 patients (27%), *Staphylococcus aureus* was identified as the causative microorganism. In two cases, the identified subspecies was methicillin-resistant *Staphylococcus aureus*. Therefore, 44 patients (26%) were included in this analysis. Among the 44 selected patients, 23 patients underwent mitral valve repair and 21 patients received mitral valve replacement (Figure 1).

Table 1 represents the preoperative baseline characteristics of the analyzed patients. The baseline demographics and clinical status of the two groups were comparable after IPTW. The mean weighted age at the time of operation was 58 years and the weighted median European System for Cardiac Operative Risk Evaluation II was 6.6. The median time between diagnosis and surgery (surgical delay) was comparable between the two groups. Most patients were treated with between one and two weeks of antibiotic therapy before surgery. Concomitant aortic and/or tricuspid valve infective endocarditis was balanced between the two groups after IPTW. The most common indication for surgery was large or embolized vegetation, especially in patients undergoing mitral valve repair.

### 3.2. Operative Characteristics

Table 2 shows the operative characteristics of the two surgical strategies. Isolated mitral valve surgery was performed in 15 patients in the repair group and in 7 patients in the replacement group, *p* = 0.07. Concomitant procedures, especially tricuspid valve surgery, were more often necessary in the replacement group. These differences led to significantly higher rates of cardiopulmonary bypass and aortic cross-clamp times in the replacement group. After IPTW, the two groups showed similar characteristics, with acceptable SMDs.

### 3.3. Outcome

All participants were followed for two years after surgery or until death. The outcome measures are presented in Table 3. Unweighted 30-day mortality was 30% in the mitral valve repair group and 38% in the mitral valve replacement group, *p* = 0.59. At two-year follow-up, the overall unweighted mortality increased to 48% in both groups (*p* = 0.99). There were no significant differences between the groups in terms of overall and event-free survival during two-year follow-up in the unweighted data. However, weighted comparison showed significant two-year overall (*p* = 0.02) and event-free survival (*p* < 0.01) benefits in the replacement group. Postoperative complications were more frequent in the mitral valve replacement group. These differences did not reach statistical significance in the unweighted comparison. In the weighted comparison, the postoperative dialysis requirement was significantly more common in the repair group.

Recurrent endocarditis occurred in three patients who were treated using mitral valve repair. The causative microorganism in all three cases was again *Staphylococcus aureus*. However, the time intervals between the index operation and relapse were 1, 8, and 12 months. The patients underwent reoperation, two re-repairs and one replacement. All three recurrent cases were alive during follow-up. In the mitral valve replacement group, no relapse or reinfection was diagnosed. One patient in the replacement group underwent reoperation due to paravalvular leakage two months after the index operation. The risk of recurrence was significantly higher in the repair group in the weighted analysis.

## 4. Discussion

*Staphylococcus aureus* is a frequent cause of infective endocarditis, associated with severe clinical presentation and a complicated course. Compared to aortic valve endocarditis, mitral valve endocarditis is significantly more often caused by *Staphylococcus aureus* [11]. Short- and long-term mortality in cardiac surgical patients with *Staphylococcus* endocarditis seems to be twice as high as in endocarditis caused by other pathogens [12]. In patients with mitral valve endocarditis caused by *Staphylococcus aureus*, the role of mitral valve repair has not been studied before. Specifically, the risk of recurrent endocarditis has been unclear. In this study, the outcomes of mitral valve repair compared to mitral valve replacement in patients undergoing surgical treatment for mitral valve infective endocarditis caused by *Staphylococcus aureus* were evaluated. First of all, operative mortality seems to be extremely high in patients with *Staphylococcus aureus* mitral valve infective endocarditis. Furthermore, mitral valve repair showed inferiority in comparison to mitral valve replacement in terms of the overall weighted mortality and event-free survival up to two years. The rates of recurrent endocarditis and mid-term mortality were significantly higher in patients undergoing mitral valve repair, leading to poorer outcomes.

The role of mitral valve repair in patients with infective endocarditis of the mitral valve has been evaluated in several studies [4,5]. Although most studies found survival benefits in patients receiving mitral valve repair, controversy surrounding recurrence rates still exists, especially in patients with *Staphylococcus* mitral endocarditis. Relapse rates of up to 10% in mitral valve repair are reported, especially when *Staphylococcus* species are the identified causative organism [13,14,15]. The virulence of *Staphylococcus aureus* is caused by its adhesion molecules that adhere to and form vegetations on the heart’s endothelial cells [16]. In addition to adhesion, *Staphylococcus aureus* is associated with large vegetations [17,18]. Furthermore, the toxins secreted by *Staphylococcus aureus* include membrane-damaging and cytolytic enterotoxins and chemotoxins [19]. These characteristics enable this pathogen to evade elimination by the host innate immune system. Therefore, the radical eradication of infected tissue is extremely important in patients undergoing cardiac surgery for infective endocarditis caused by *Staphylococcus aureus*. Therefore, the radical resection of infected valve tissue should be performed to prevent recurrence.

The incidence of *Staphylococcus aureus* infective endocarditis of the native valve is increasing [20]. The median age in this population is significantly lower than in native valve infective endocarditis caused by other pathogens. Furthermore, patients with *Staphylococcus aureus* infective endocarditis are less likely to undergo surgery compared to patients with endocarditis caused by other microorganisms [21], resulting in significantly higher mortality rates [22,23]. Finally, mitral valve involvement [24] and the associated increased mortality [25] of *Staphylococcus aureus* infective endocarditis have been noted and confirmed in our current study.

The increased risk of recurrent endocarditis in mitral valve repair in the current study could be explained by several factors. The delays between diagnosis and surgery in the three patients who developed recurrent endocarditis were 3, 4, and 9 days on antibiotic treatment at the time of index operation. Although early surgery is associated with improved short- and mid-term outcomes [26,27], a higher risk of recurrence and valvular dysfunction has been reported in patients receiving less than one week of antibiotic therapy before surgery [28]. Also, postoperative combination antibiotic therapy seems to increase the risk of recurrence compared to monotherapy [29]. In the current analysis, the included patients were treated with both combination and monotherapy. Furthermore, a maximum effort towards repair was applied for these patients due to their young age [30]. The risk factor associated with recurrent endocarditis [31,32] was present in all three recurrence cases (two intravenous drug users and one patient with poor dental hygiene). Finally, patients undergoing mitral valve repair more often presented with large or embolized vegetations. Large vegetations have been shown not only to increase the risk of embolism, but also in-hospital mortality [33,34].

## 5. Limitations

To the best of our knowledge, the current study is the first study comparing mitral valve repair to mitral valve replacement in patients with native mitral valve infective endocarditis due to *Staphylococcus aureus*. Although the baseline and operative characteristics between the two groups were comparable after IPTW, this study retained a small sample size and retrospective and non-randomized analysis with associated bias. The lack of information on the pathoanatomic severity of the disease, specific surgical techniques applied during the operations, and echocardiographic follow-up are important limitations of the current analysis. We therefore acknowledge that our findings are exploratory and hypothesis-generating. Definitive conclusions cannot be drawn from this analysis. Prospective, randomized control trials with a large sample size are needed to evaluate the role of mitral valve repair in patients with mitral valve infective endocarditis due to *Staphylococcus aureus*.

## Figures and Tables

**Table 1:** Preoperative characteristics.

	Unweighted				Weighted			
Variables	Repair N = 23	Replacement N = 21	*p*	SMD	Repair N = 42	Replacement N = 41	*p*	SMD
**Demographics**								
Age, *years ± SD*	60 ± 14	59 ± 11	0.95	0.018	59 ± 12	57 ± 10	0.52	0.141
Dialysis, *n (%)*	4 (17)	5 (24)	0.72	0.079	6 (14)	7 (17)	0.73	0.038
Previous cardiac surgery, *n (%)*	1 (4)	3 (14)	0.34	0.173	1 (2)	3 (7)	0.36	0.115
EuroSCORE II, *% (IQR)*	4.5 (3.1–12.1)	6.7 (3.1–27.9)	>0.99	0.459	7.6 (3.2–12.2)	4.2 (2.3–25.7)	0.07	0.186
**Clinical status**								
Preop. intubated, *n (%)*	4 (17)	7 (30)	0.22	0.184	7 (17)	10 (24)	0.39	0.094
Preop. vasopressor need, *n (%)*	3 (13)	5 (24)	0.45	0.139	5 (12)	7 (17)	0.50	0.073
Surgical delay, *days (IQR)*	10 (6–15)	7 (5–17)	0.75	0.022	8 (4–14)	6 (4–14)	0.50	0.050
**Echocardiographic parameters**								
Severe MR, *n (%)*	11 (48)	8 (38)	0.52	0.098	17 (40)	18 (44)	0.83	0.024
Concomitant AV IE, *n (%)*	3 (13)	6 (29)	0.27	0.192	15 (36)	11 (27)	0.38	0.096
Concomitant TV IE, *n (%)*	2 (9)	3 (14)	0.66	0.088	3 (7)	4 (10)	0.67	0.046
**Indication for surgery**								
Heart failure, *n (%)*	4 (17)	4 (19)	>0.99	0.021	7 (17)	8 (20)	0.70	0.042
Large/embolized vegetation, *n (%)*	15 (65)	8 (38)	0.07	0.271	28 (67)	18 (44)	0.05	0.213
Uncontrolled infection, *n (%)*	3 (13)	8 (38)	0.06	0.289	6 (14)	10 (24)	0.24	0.128
Valvular disease, *n (%)*	1 (4)	1 (5)	>0.99	0.010	2 (5)	5 (12)	0.26	0.136

Data are presented as mean ± standard deviation or median (interquartile range), number (percentage) and standardized mean difference (SMD); EuroSCORE, European System for Cardiac Operative Risk Evaluation; MR, mitral regurgitation; AV, aortic valve; TV, tricuspid valve; and IE, infective endocarditis.

**Table 2:** Intraoperative findings and procedures performed.

	Unweighted				Weighted			
Variables	Repair N = 23	Replacement N = 21	*p*	SMD	Repair N = 42	Replacement N = 41	*p*	SMD
Isolated MV surgery, *n (%)*	14 (61)	7 (33)	0.07	0.275	19 (45)	18 (44)	0.90	0.013
Concomitant CABG, *n (%)*	3 (13)	4 (19)	0.69	0.082	13 (31)	8 (20)	0.26	0.124
Concomitant AV surgery, *n (%)*	5 (22)	7 (33)	0.39	0.130	18 (43)	12 (29)	0.22	0.131
Concomitant TV surgery, *n (%)*	2 (9)	6 (29)	0.13	0.257	3 (7)	7 (17)	0.20	0.150
CPB time, *minutes (IQR)*	83 (73–122)	126 (114–147)	<0.01	0.612	105 (76–204)	120 (93–145)	>0.99	0.112
ACC time, *minutes (IQR)*	53 (42–78)	86 (76–102)	0.02	0.830	77 (46–133)	84 (76–97)	0.26	0.161

Data are presented as number (percentage) or median (interquartile range) and standardized mean difference (SMD); MV, mitral valve; CABG, coronary artery bypass grafting; AV, aortic valve; TV, tricuspid valve; CPB, cardiopulmonary bypass; and ACC, aortic cross-clamp.

**Table 3:** Endpoints.

	Unweighted				Weighted			
Variables	Repair N = 23	Replace N = 21	*p*	OR (95% CI)	Repair N = 42	Replace N = 41	*p*	OR (95% CI)
**Primary**								
Mortality (30-day), *n (%)*	7 (30)	8 (38)	0.59	0.84 (0.45–4.91)	18 (43)	11 (27)	0.15	1.44 (0.85–2.43)
Mortality (2-year), *n (%)*	11 (48)	10 (48)	0.99	1.00 (0.54–1.87)	24 (57)	13 (32)	0.02	1.73 (1.06–2.84)
MV recurrent IE (2-year), *n (%)*	3 (13)	-	0.23	NA	6 (14)	-	0.03	NA
MV reoperation (2-year), *n (%)*	3 (13)	1 (5)	0.61	2.00 (0.36–11.23)	6 (14)	4 (10)	0.74	1.25 (0.57–2.76)
Event-free survival (2-year), *n (%)*	9 (39)	10 (48)	0.57	0.85 (0.47–1.52)	12 (29)	24 (59)	<0.01	0.52 (0.31–0.87)
**Secondary**								
Revision for bleeding, *n (%)*	1 (4)	3 (14)	0.34	0.60 (0.31–1.16)	2 (5)	3 (7)	0.67	0.80 (0.38–1.70)
Stroke, *n (%)*	-	1 (5)	0.48	NA	-	3 (7)	0.12	NA
MCS, *n (%)*	1 (4)	2 (10)	0.60	0.70 (0.29–1.65)	2 (5)	2 (5)	>0.99	0.98 (0.36–2.67)
Reintubation, *n (%)*	5 (22)	10 (48)	0.09	1.82 (0.84–3.95)	8 (19)	15 (37)	0.09	0.68 (0.45–1.02)
Dialysis, *n (%)*	4 (17)	3 (14)	>0.99	1.14 (0.45–2.84)	15 (36)	3 (7)	<0.01	3.46 (1.21–9.91)
ICU stay, *days (IQR)*	5 (3–7)	8 (4–19)	0.10	NA	4 (1–7)	7 (4–14)	0.07	NA
Hospital stay, *days (IQR)*	8 (6–14)	10 (7–18)	0.77	NA	8 (3–11)	9 (7–19)	0.36	NA

Data are presented as number (percentage) or median (interquartile range) and odds ratio (95% confidence interval); MV, mitral valve; IE, infective endocarditis; MCS, mechanical circulatory support; ICU, intensive care unit; and NA, not applicable.

## Data Availability

The data underlying this article will be shared upon reasonable request to the corresponding author.
